# Preceptor-Ranked Competencies in Osteopathic Medical Students: A Comparison of General Surgery and Surgical Subspecialties

**DOI:** 10.7759/cureus.95657

**Published:** 2025-10-29

**Authors:** Ryan D Muchard, Trevor J Donnelly, Cole S Arnold, Rahul Garg, Richard R Thacker, Sherry L Roach

**Affiliations:** 1 Clinical Sciences, Alabama College of Osteopathic Medicine, Dothan, USA; 2 Surgey, SSM St. Louis University, St. Louis, USA

**Keywords:** clinical clerkship, clinical competencies, clinical rotation, medical education, medical student evaluation, preceptor expectations, residency preparation, surgical clerkship, surgical education, surgical subspecialty

## Abstract

Introduction

As the Comprehensive Osteopathic Medical Licensing Examination (COMLEX) Level 1 and United States Medical Licensing Exam (USMLE) Step 1 have transitioned to pass/fail scoring, greater emphasis has been placed on clinical performance and subjective evaluations during surgical clerkships. While prior work explored preceptor expectations in general surgery, the competencies valued across surgical subspecialties remain less defined. This study aims to build on previous work by increasing the sample size and identifying the qualities and skills that surgical preceptors most value in medical students across both general surgery and surgical subspecialty disciplines.

Methods

A descriptive cross-sectional survey study was conducted among surgical preceptors within the faculty network at the Alabama College of Osteopathic Medicine (ACOM). A 10-item web-based survey, utilizing the Qualtrics XM platform (Qualtrics, North Sydney, Australia), was adapted from previous published work on general surgeons and distributed to additional surgery preceptors (Doctor of Medicine (MD) and Doctor of Osteopathic Medicine (DO)). The survey captured data on six core competency domains and their respective subcomponents. Responses from general surgery preceptors (n = 25) were combined with newly collected responses from additional general surgeons and surgical subspecialty preceptors (n = 39), resulting in a total sample of 64. Descriptive and comparative analyses were performed to identify both shared and specialty-specific preferences.

Results

Across all specialties, nontechnical attributes were prioritized over procedural skills. Ranking questions were quantified using one-to-six or one-to-five scales so that higher values represented greater perceived importance (0 = least important; 5 or 6 = most important). Enthusiasm and willingness to learn (mean = 4.1), differential diagnosis (mean = 3.7), and anatomic knowledge (mean = 3.5) were rated highest, while leadership and deep suturing were consistently ranked lowest. Differences were observed between specialties for operating room knowledge and knot tying and suturing. Orthopedic preceptors rated deep suturing higher (mean = 1.3), while general surgeons valued hand ties more (mean = 2.9). Notably, 51% of preceptors reported adjusting expectations based on a student’s intended specialty.

Conclusion

Surgical preceptors across specialties consistently valued enthusiasm, adaptability, and clinical reasoning over early technical skills. These findings highlight the need for medical students to prioritize interpersonal and cognitive competencies while gradually developing procedural abilities. Specialty-specific differences underscore the importance of tailored preparation, which may improve clerkship performance, evaluations, and residency readiness.

## Introduction

As the landscape of surgical residency applications continues to evolve, osteopathic and allopathic medical students are challenged to demonstrate more than academic proficiency; they must cultivate the specific qualities, knowledge, and skills that surgical preceptors recognize as markers of exceptional clinical potential. The transition of the Comprehensive Osteopathic Medical Licensing Examination (COMLEX) Level 1 and the United States Medical Licensing Exam (USMLE) Step 1 to pass/fail scoring has heightened the significance of subjective evaluations such as clerkship performance, sub-internship assessments, and letters of recommendation [1-2]. These elements now serve as key differentiators in the residency selection process, particularly in high-stakes specialties like surgery, where students must perform under pressure, adapt to complex environments, and collaborate effectively within the operating room. In this setting, aligning with preceptor expectations is critical to success, yet those expectations often remain unclear, leaving students without concrete guidance on how to stand out [3-4].

Our previous study focused on general surgery preceptors and identified that nontechnical attributes, such as enthusiasm, receptiveness to feedback, and clinical reasoning, were often ranked above technical skills [5]. However, feedback from that work underscored the need for a broader perspective. This follow-up study was designed to attempt to fill that gap. By surveying a wider range of surgical preceptors, including those in urology, orthopedics, plastics, neurosurgery, trauma, vascular, cardiothoracic, and bariatric surgery, this study aims to clarify how subspecialty preceptors evaluate competencies such as suturing proficiency, operating room etiquette, and preclinical medical knowledge.

Understanding these preferences is especially vital for students navigating surgical clerkships and subinternships, as misalignment between student performance and preceptor expectations can lead to missed opportunities and underwhelming evaluations. While competency-based education provides a broad framework for clinical growth, it often lacks real-world granularity. Preceptors assess students not only on knowledge and technical ability but also on teamwork, adaptability, and eagerness to learn-qualities that are rarely emphasized in preclinical education and often acquired only through direct experience [6-7]. This disconnect can leave students feeling underprepared, particularly in the fast-paced, high-stakes surgical environment [7].

With residency programs placing greater weight on clerkship grades and subjective evaluations in the absence of a numeric Step 1 or Level 1 score, students must be increasingly strategic in how they prepare for and perform during clinical training [8]. By identifying the specific qualities and competencies surgical preceptors value most, both universally and within individual specialties, this study aims to provide medical students with practical guidance to optimize their clinical performance, strengthen their evaluations, and increase their competitiveness for surgical residency positions. This article was previously presented as an abstract and poster at the 2023 American College of Osteopathic Surgeons (ACOS) Annual Clinical Assembly, Chicago, Illinois, September 22, 2023.

## Materials and methods

**Figure 1:**
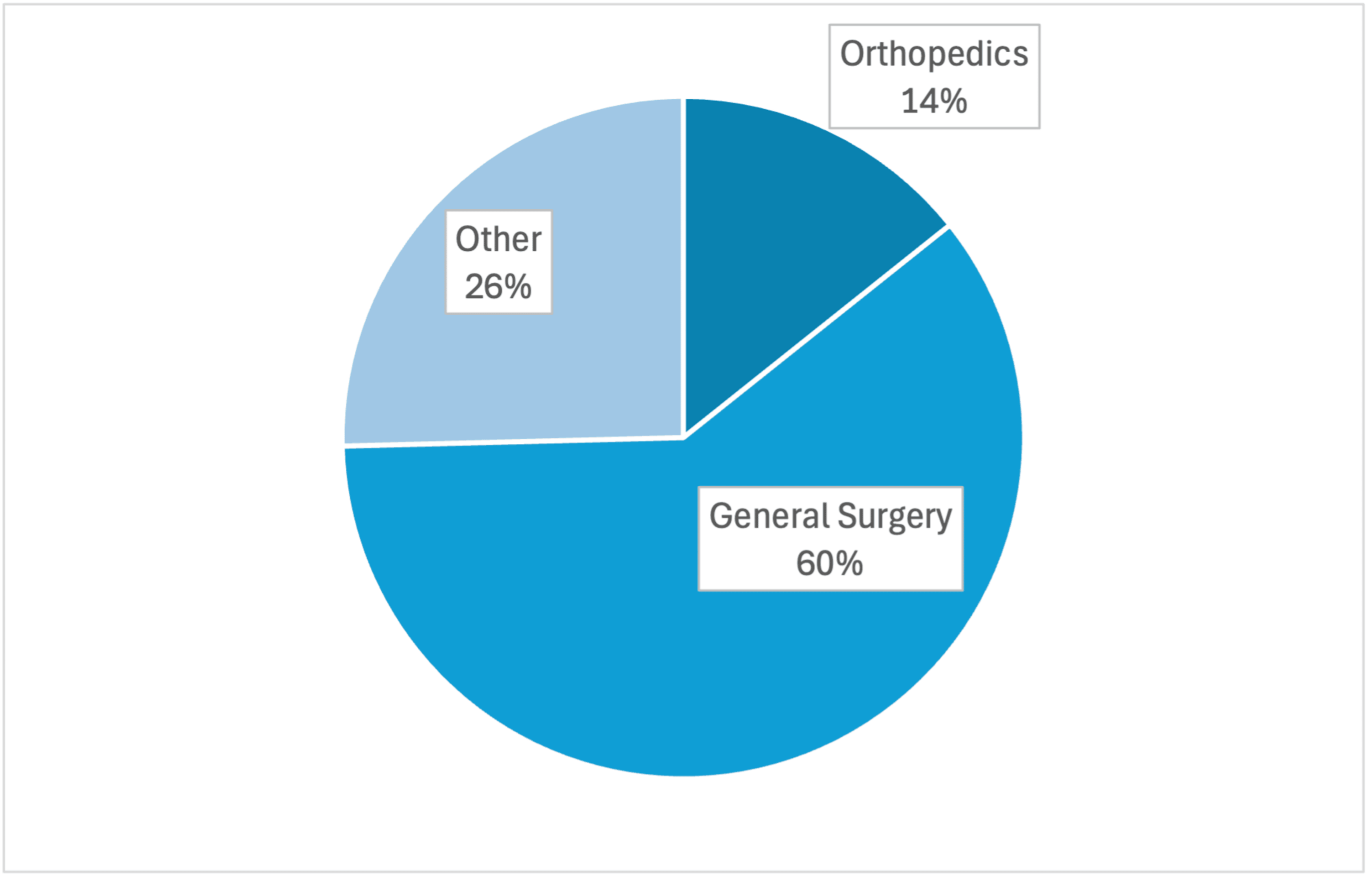
Surgical subspecialties surveyed Note. Other subspecialties include urology, plastic, neurosurgery, trauma, vascular, cardiothoracic, and bariatric. n = 64

A descriptive, cross-sectional survey study initially published for general surgery was adapted to explore surgical preceptor perspectives on the competencies, characteristics, and clinical skills most valued in exceptional medical students. A 10-question web-based survey was developed using the Qualtrics XM platform (Qualtrics, North Sydney, Australia). Survey distribution occurred over a three-month period, with three reminder emails sent at three-week intervals to 211 preceptors. All responses were collected anonymously. A total of 39 complete responses were received, resulting in a response rate of 18.5%. Responses collected were from surgical subspecialty preceptors and additional general surgeons not included in the original study. This was combined with 25 previously collected responses from general surgery preceptors, yielding a combined dataset of 64 responses (n = 64). All participants were within the Alabama College of Osteopathic Medicine (ACOM) clinical network across Alabama, Florida, Georgia, and Mississippi, including physicians from general surgery and a variety of surgical subspecialties (e.g., orthopedic, urology, plastic, neurosurgery, trauma, vascular, cardiothoracic, and bariatrics (Figure 1). Eligible participants included attending physicians with Doctor of Osteopathic Medicine (DO) or Doctor of Medicine (MD) degrees who had previously served as preceptors for third- or fourth-year medical students.

**Figure 2:**
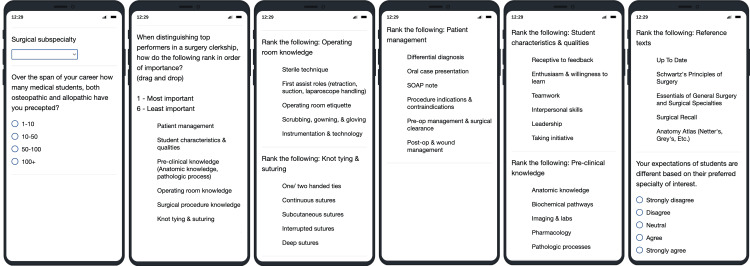
Survey questions Multiple choice (questions 1-2). Ranking drag and drop (questions 3-9): 1, most important; 6, least important (data inverted for interpretation in figures). Likert scale (question 10)

The survey was designed to assess preceptor perspectives and identify attributes and skills associated with osteopathic student success in surgical rotations. It included a combination of multiple-choice, ranking, and Likert-scale questions. Ranking questions were collected on either a 1-6 or a 1-5 scale. For consistency, responses were recoded so that the most important rank received the highest numerical value. Specifically, scores were inverted and shifted to a 0-6 scale, such that an original rank of 1 (most important) was recoded as 6, and the lowest rank on the original scale was recoded as 0. This ensured that higher values uniformly indicate greater perceived importance across all figures. The third item asked preceptors to rank 6 broad domains of student core competencies, such as professional characteristics, operating room knowledge, and clinical decision-making, based on perceived importance. The subsequent questions broke these domains into subcategories (e.g., enthusiasm, suturing techniques, anatomic knowledge) to allow for a more granular analysis (Figure 2).

Descriptive statistics, including mean values, were used to assess the relative importance of each competency and its corresponding subcompetencies. Higher mean values reflected higher perceived importance. For ease of conducting descriptive comparative analysis, three groups were formed. General surgeons, orthopedics, and other subspecialties. A comparative analysis was conducted using Kruskal-Wallis and Analysis of Variance (ANOVA) tests to assess differences in preceptor preferences across surgical subspecialties. However, due to the low sample size and the use of mean values, these inferential statistical tests were omitted for concerns regarding validity.

This structured approach enabled identification of both shared and specialty-specific expectations, offering practical insights into surgical preceptor priorities that may inform student preparation and educational curricula.

## Results

**Figure 3:**
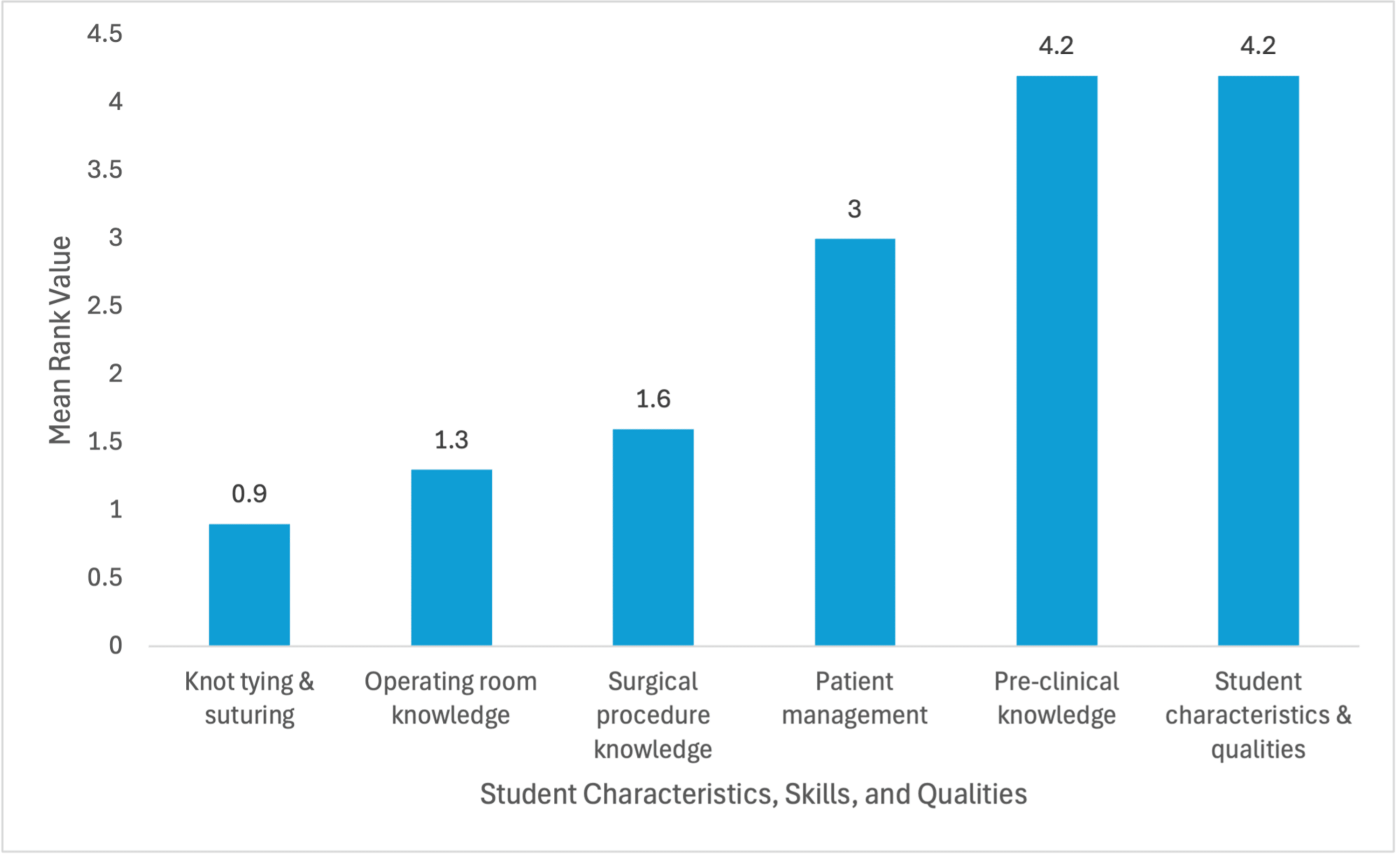
Student skills, characteristics, and qualities valued the most by all surgical preceptors 0, least valued; 6, most valued

Survey responses were completed in full by 39 of the recipients (n = 39), including preceptors from general surgery, orthopedics, and a range of surgical subspecialties. These were combined with previously published responses from 25 general surgery preceptors, resulting in a total dataset of 64 complete responses (n = 64). General surgeons (n = 38), orthopedics (n = 9), and other subspecialties (n = 16) made up of two bariatric, two trauma, three plastics, one cardiothoracic, two vascular, one neurosurgery, one urology, two micrographic, and two in the other category. The one response that did not specify their specialty was omitted from the comparative analysis but included in the overall data analysis. Preceptors ranked core competencies from least to most important, with a higher mean value indicating more importance. Student characteristics and qualities (mean = 4.2), preclinical knowledge (mean = 4.2), and patient management (mean = 3.0) received the highest overall values. Surgical procedure knowledge (mean = 1.6), operating room knowledge (mean = 1.3), and knot tying and suturing (mean = 0.9) were ranked as having lower perceived importance (Figure 3).

Subcategory analysis revealed additional trends within each core competency. Among student characteristics, enthusiasm and willingness to learn (mean = 4.1) were rated as the highest, while leadership was rated as the least valued (mean = 0.6). For preclinical knowledge, anatomic knowledge (mean = 3.5) and understanding of pathologic processes (mean = 3.2) were prioritized over pharmacology (mean = 1.0) and biochemical pathways (mean = 0.6). In the domain of patient management, differential diagnosis (mean = 3.7) and oral case presentation (mean = 3.0) were considered more valuable than postoperative or wound care management (mean = 1.2). Surgical procedure knowledge, as ranked by general surgeons, valued appendectomy (mean = 3.0) and cholecystectomy (mean = 2.7) as the highest among the listed procedures. Within operating room knowledge, sterile technique (mean = 3.2) was valued most, while instrumentation and technology (mean = 0.6) were rated lowest. For knot tying and suturing, interrupted sutures (mean = 3.1) and one- or two-handed ties (mean = 2.5) were prioritized over deep sutures (mean = 0.6) (Table 1).

**Table 1:** Ranked student skills, characteristics, and qualities by all surgical specialty preceptors SOAP: subjective, objective, assessment, and plan. 0, least important

Rank order results	Mean
Student characteristics	
Enthusiasm & willingness to learn	4.1
Interpersonal skills	3.0
Taking initiative	2.6
Receptive to feedback	2.3
Teamwork	2.3
Leadership	0.6
Preclinical knowledge	
Anatomic knowledge	3.5
Pathologic process	3.2
Imaging & labs	1.8
Pharmacology	1.0
Biochemical pathways	0.6
Patient management	
Differential diagnosis	3.7
Oral case presentation	3.0
Procedure indications & contraindications	2.8
Preop management	2.1
SOAP note	2.1
Postop/wound management	1.2
Surgical procedure knowledge (general surgery specific)	
Appendectomy	3.0
Cholecystectomy	2.7
Herniorrhaphy	1.9
Breast biopsy & mastectomy	1.4
Colectomy & small bowel repair	1.0
Operating room knowledge	
Sterile technique	3.2
Scrubbing, gowning, gloving	2.6
Operating room etiquette	2.5
First assist roles (retraction, suction)	1.1
Instrumentation/technology	0.6
Knot tying & suturing	
Interrupted sutures	3.1
One-/two-handed ties	2.5
Subcutaneous sutures	2.0
Continuous sutures	1.9
Deep sutures	0.6

**Figure 4:**
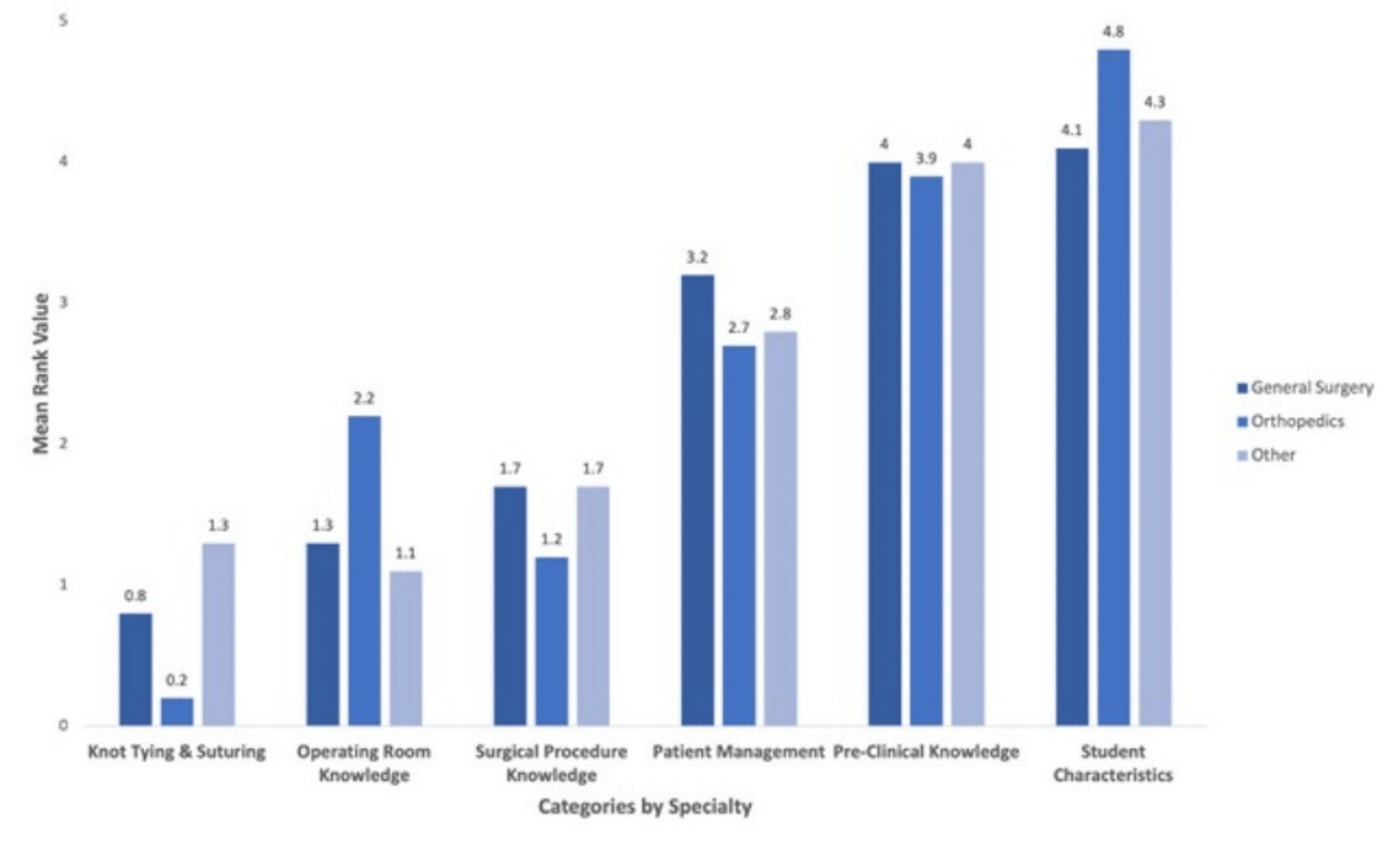
Student skills, characteristics, and qualities valued the most by surgical subspecialty preceptors 0, least valued; 6, most valued

**Figure 5:**
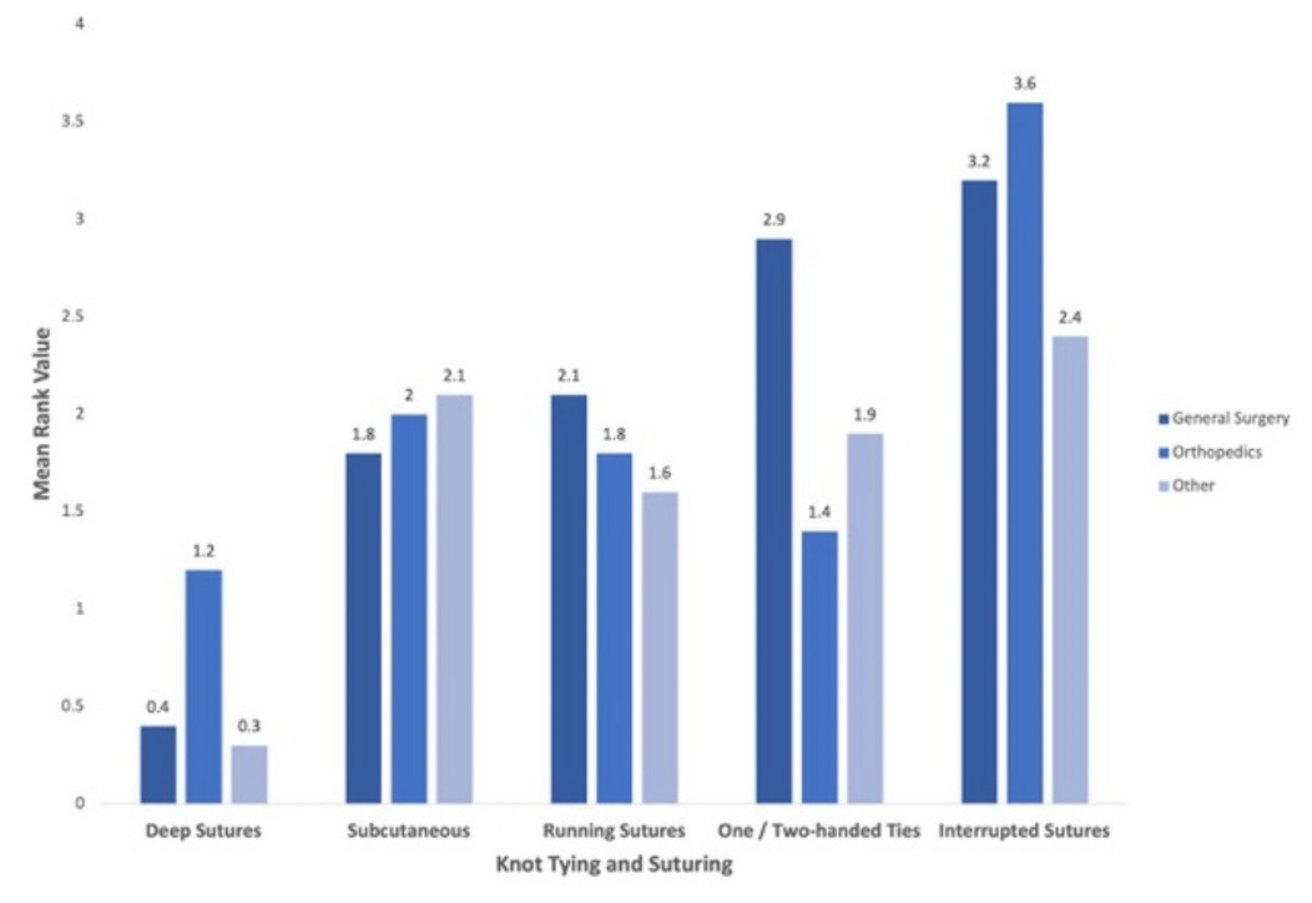
Student skills in knot tying and suturing valued the most by surgical subspecialty preceptors 0, least valued; 5, most valued

Stratification of results by surgical discipline (general surgery, orthopedics, and other subspecialties) revealed differences in perceptions of operating room knowledge and knot tying and suturing. The rank for operating room knowledge varied across specialties: general surgery (mean = 1.3), orthopedics (mean = 2.2), and other subspecialties (mean = 1.1). Knot tying and suturing also demonstrated variation: general surgeons (mean = 0.8), orthopedic surgeons (mean = 0.2), and other specialties (mean = 1.3). The remaining categories displayed minimal differences by specialty (Figure 4). Further subanalysis of knot-tying techniques showed that orthopedic preceptors rated deep suturing more highly (mean = 1.3) compared to general surgery (mean = 0.4) and other subspecialties (mean = 0.3). In contrast, general surgeons placed a higher value on one- and two-handed ties (mean = 2.9), followed by other specialties (mean = 1.9) and orthopedic surgeons (mean = 1.4). The remaining suturing techniques showed less variation by specialty (Figure 5).

**Figure 6:**
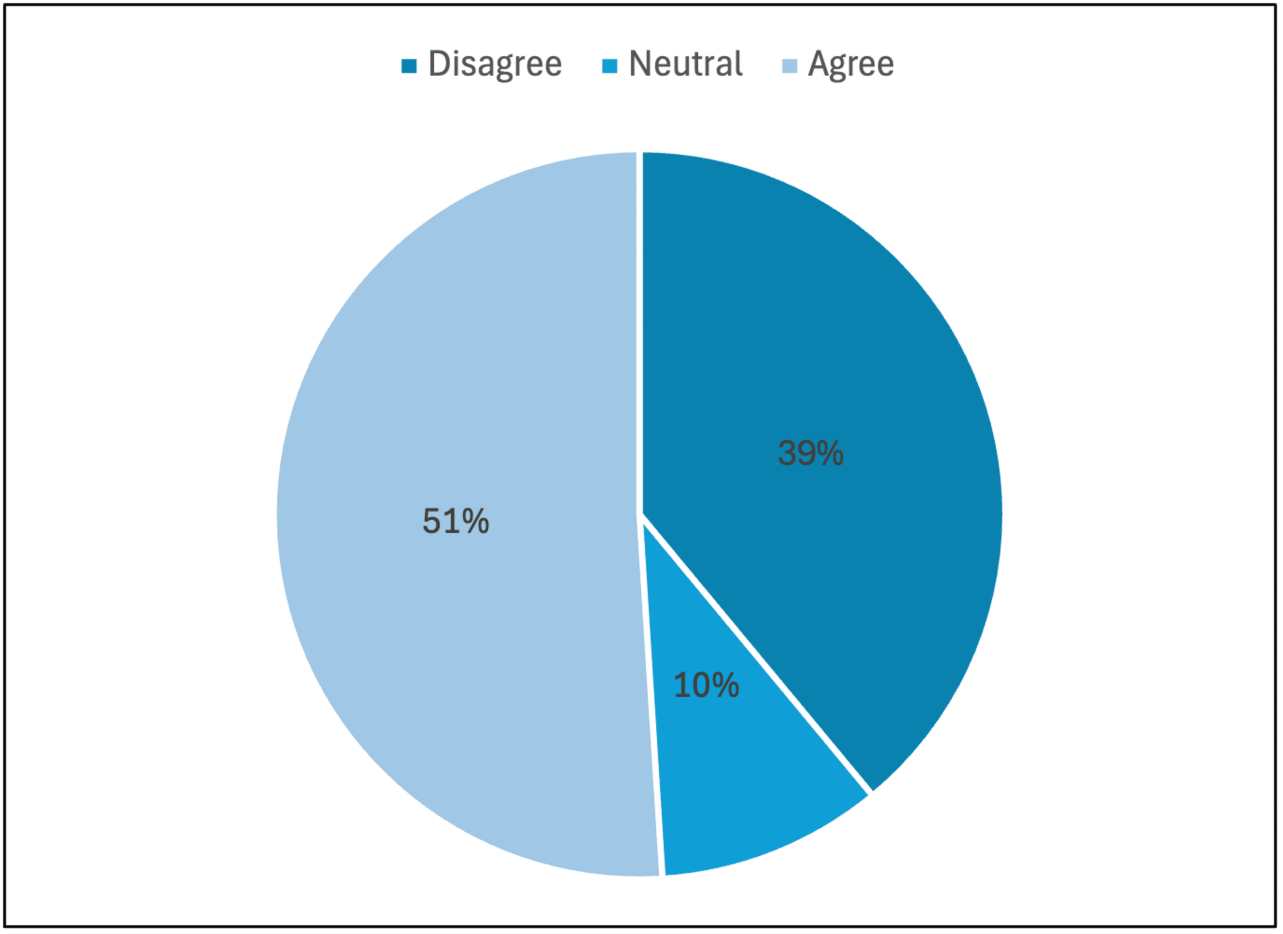
"Your expectations of students are different based on their specialty of interest"

In the final question of the survey, 51% of surgical preceptors agreed that their expectations of students differ based on the students’ specialty of interest, while 10% remained neutral and 39% disagreed (Figure 6). There were no significant differences based on specialty.

## Discussion

This expanded study builds on previous work examining general surgery preceptor expectations by incorporating responses from eight additional surgical subspecialties. Together, the findings underscore a consistent prioritization of nontechnical attributes, particularly enthusiasm, receptiveness to feedback, and clinical reasoning, over early technical skills. These preferences reflect an evolving recognition within surgical education that interpersonal qualities, adaptability, and situational awareness form the foundation for successful clinical performance [9-13]. Prior studies similarly report that both students and faculty emphasize social-interactive competencies, professionalism, and motivation as critical to success during surgical rotations [9-13].

While the importance of technical skills is undeniable, this study reinforces that early surgical learners are often evaluated more on their attitudes and work habits than on procedural ability. In both general surgery and subspecialties, preceptors consistently valued enthusiasm and a willingness to learn more highly than knot-tying, suturing, or advanced procedural knowledge. Interestingly, specialty-specific differences were observed: orthopedic preceptors placed greater emphasis on deep suturing, whereas general surgeons prioritized hand ties. These findings highlight the procedural heterogeneity within surgical education and support the need for competency-based curricula tailored to the demands of each specialty [14,15].

This approach is increasingly supported by the adoption of entrustable professional activities (EPAs) and milestones across U.S. medical education, which aim to integrate both technical and nontechnical skills into progressive, skills-based training and assessment models [15,16]. Such frameworks promote individualized readiness over time-based progression, encouraging educators to focus on qualities like diagnostic reasoning, communication, and receptiveness that can be observed and nurtured even in early rotations.

A noteworthy finding was that 51% of surveyed preceptors acknowledged adjusting expectations based on a student’s stated specialty interest. While this may represent a pragmatic approach, aligning feedback and opportunities with perceived career trajectories, it also raises important considerations. Many students modify their specialty preferences during clinical training, and variable expectations could inadvertently skew their educational experiences, potentially influencing final career choices [17]. This highlights the need for a structured orientation, clear expectations, and consistent evaluation criteria, regardless of a student's interest.

The study’s broader implications for preclinical medical education include a call to action for early intervention. Structured preparation strategies, such as preclerkship skills boot camps, surgical shadowing, and specialty-specific orientation sessions, may better align student readiness with preceptor expectations [14,16,18]. Importantly, this preparation should not focus solely on procedural training but should also emphasize the interpersonal and cognitive skills that preceptors consistently value.

Previous literature has similarly emphasized that emotional intelligence, humility, confidence, and collaboration have a significant impact on clinical performance and student-preceptor relationships [19-22]. Our findings reinforce this, demonstrating that enthusiasm and a proactive attitude ranked above leadership or procedural knowledge across specialties. While leadership remains a key goal for residency training, early student success appears more closely tied to curiosity, humility, and adaptability [22]. Medical education programs that cultivate resilience, self-directed learning, and receptiveness may empower students to become more effective contributors in surgical settings [23].

As the field continues to shift toward competency-based assessment, the role of the educator has expanded. Faculty are now expected to promote psychological safety, foster learner-centered environments, and adapt to a variety of student needs [24]. Nontechnical skills are being increasingly integrated into surgical training through tools such as the nontechnical skills for surgeons (NOTSS) framework and structured feedback models [25]. These strategies reflect the growing understanding that cognitive and interpersonal readiness often precede and predict the successful acquisition of technical expertise.

Finally, as surgical training becomes more global and diverse, the importance of interpersonal effectiveness, communication, and adaptability is further emphasized. Studies exploring the competencies valued in international medical graduates and across diverse educational backgrounds underscore the need for standardized, equitable evaluation systems that focus on nontechnical attributes [26,27]. By continuing to prioritize these skills in undergraduate education, surgical educators can better identify and support students with strong potential, regardless of background, while also fostering an inclusive and growth-oriented learning culture.

Limitations

Limitations of this study include the modest sample size and potential response bias, as preceptors with strong opinions may be more likely to participate. The descriptive cross-sectional design precludes assessment of causality or longitudinal changes in preferences. The study was conducted within a single osteopathic medical college faculty network, which may not capture the full spectrum of surgical subspecialty perspectives or institutional variations in educational culture. Multiple statistical comparisons were conducted without formal correction, as the study was exploratory in nature; thus, significant p-values were not included out of caution due to the increased risk of type 1 error. Finally, while the survey results provide valuable insights, further studies with larger, multi-institutional cohorts and qualitative methodologies may be needed to confirm these findings and explore additional perspectives.

## Conclusions

This study highlights that surgical preceptors across general surgery and subspecialties consistently prioritize enthusiasm, adaptability, and clinical reasoning over technical proficiency in early learners. These findings carry important clinical implications for both students and educators. For students, success during surgical clerkships and subinternships may depend less on arriving with polished procedural skills and more on demonstrating curiosity, receptiveness to feedback, and strong foundational knowledge. Traits that directly translate into improved evaluations and stronger residency applications. For educators and curriculum designers, the results underscore the need to implement early interventions such as preclerkship skills boot camps, structured orientation sessions, and competency-based feedback systems that emphasize interpersonal and cognitive skills alongside technical training. Specialty-specific differences, such as orthopedic preceptors valuing deep suturing and general surgeons prioritizing hand ties, further suggest that tailored preparation may enhance performance and student-preceptor alignment. As residency selection increasingly relies on subjective assessments in the absence of Step 1 or Level 1 numeric scores, bridging the gap between student preparation and preceptor expectations is critical. By cultivating the nontechnical skills most valued across surgical disciplines while integrating specialty-specific procedural training, medical education programs can better equip osteopathic medical students to excel in surgical environments and ultimately improve their readiness for residency.

## Figures and Tables

**Figure 7:**
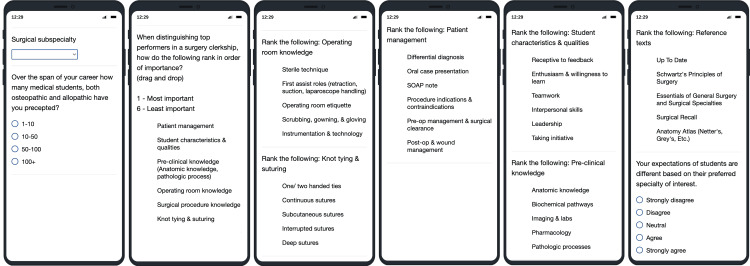
Full survey questionaire
